# Serum Levels of 18 Trace Elements and Type 2 Diabetes Mellitus: A Propensity Score-Matched Case–Control Study in Northwest Chinese Adults

**DOI:** 10.3390/nu18183068

**Published:** 2026-09-20

**Authors:** Lu Ma, Xianglong Liu, Boqian Feng, Yu Zhao, Jiaxing Zhang, Yi Zhao, Ling Fan

**Affiliations:** 1 School of Public Health, Ningxia Medical University, No. 1160 Shengli Street, Xingqing District, Yinchuan 750004, China; ml18328537511@163.com (L.M.); 18195136912@163.com (X.L.); 15009699029@163.com (B.F.); zhaoyu@nxmu.edu.cn (Y.Z.); nxmu_zjx1106@163.com (J.Z.); 2 Key Laboratory of Environmental Factors and Chronic Disease Control, Ningxia Medical University, Yinchuan 750004, China

**Keywords:** type 2 diabetes mellitus, trace elements, least absolute shrinkage and selection operator, Bayesian kernel machine regression, restricted cubic splines

## Abstract

**Objective**: Current evidence on the association between multiple trace elements and type 2 diabetes mellitus (T2DM) remains limited, particularly regarding combined effects, nonlinear dose–response patterns, and elemental interactions. This study aimed to evaluate the associations between multiple serum trace elements and T2DM using a sequential analytical strategy. **Methods**: A 1:1 propensity score–matched case–control study based on sex, age, and residence was conducted (637 controls, 637 T2DM cases). Serum concentrations of 18 trace elements were measured using inductively coupled plasma mass spectrometry (ICP-MS). A sequential analytical strategy was applied: LASSO regression for variable selection; logistic regression with single-element (Model 1) and multi-element (Models 2 and 3) approaches for association assessment; weighted quantile sum (WQS) regression and Bayesian kernel machine regression (BKMR) for mixture effects; and restricted cubic spline (RCS) for dose–response patterns. **Results**: Lithium (Li) and tellurium (Te) showed positive associations with T2DM across multiple analytical methods, whereas boron (B), lead (Pb), and vanadium (V) showed inverse associations. WQS identified V and B as the main contributing elements. BKMR did not identify a statistically significant overall association between the mixture and T2DM. Although bivariate interaction plots suggested possible effect modification between V and Al, B, Li, Pb, and Te, formal interaction tests did not reach statistical significance. RCS showed that serum B was significantly associated with T2DM overall and exhibited a significant non-linear dose–response relationship (P-nonlinear = 0.0028). Serum V showed a linear inverse association with T2DM, and serum Li showed a linear positive association. **Conclusions**: Our findings suggest that Li, Te, B, and V are associated with T2DM, with the most robust evidence for Li, while the evidence for Te was less consistent. In addition, we observed a trend toward a joint association of the trace element mixture, potential effect modification at the point-estimate level between V and Al, B, Li, Pb, and Te, and distinct dose–response patterns for B, Li, and V. These findings highlight the need for further investigation of trace elements in relation to T2DM, with consideration of both individual and combined associations; future validation of these exploratory findings in larger prospective studies and independent populations is warranted.

## 1. Introduction

Diabetes mellitus is a metabolic disease characterized by hyperglycemia, pancreatic β-cell dysfunction, and peripheral insulin resistance [1]. Among its various types, type 2 diabetes mellitus (T2DM) is the most prevalent form [2,3]. Globally, 589 million adults had diabetes in 2024 (over 90% T2DM), projected to reach 853 million by 2050; China has the largest diabetic population (~148 million). Chronic hyperglycemia damages blood vessels, affecting the eyes, kidneys, heart, and nerves, and leads to diverse complications [4].

Beyond these clinical consequences, identifying modifiable risk factors is essential for prevention. In addition to well-established risk factors for T2DM such as age, obesity, unhealthy lifestyle, genetic predisposition, and family history, trace elements have been increasingly linked to T2DM [5]. Certain elements—including selenium (Se), nickel (Ni), zinc (Zn), copper (Cu), manganese (Mn), and vanadium (V)—are involved in glucose homeostasis, oxidative stress, and insulin secretion [6]. For example, Se levels have been associated with T2DM, with evidence of non-linearity [7,8]. However, most previous research has concentrated on a limited set of conventional trace elements, leaving the roles of many other elements poorly characterized. Furthermore, the vast majority of studies have examined single elements in isolation, despite the fact that humans are exposed to multiple trace elements simultaneously in daily life. The combined associations of multiple serum trace elements may differ from the independent associations of any single trace element [9]. Indeed, low levels of an individual trace element may not be harmful alone, but when combined with others, could increase disease risk [10]. Given this realistic scenario in which multiple trace elements commonly co-occur, evaluating the joint associations of multiple trace elements with T2DM is clinically meaningful, yet relevant studies remain insufficient. Although the associations between individual trace elements and T2DM have been investigated in previous studies, most have focused on specific categories of elements using single-element regression models that cannot account for joint effects, non-linear patterns, or elemental interactions.

The present study aimed to comprehensively investigate the associations between multiple trace elements and T2DM through a sequential analytical strategy. First, we applied Least Absolute Shrinkage and Selection Operator (LASSO) regression to screen for key trace elements most strongly associated with T2DM while mitigating multicollinearity. Second, logistic regression assessed individual associations and evaluated whether the associations remained after adjustment for coexisting elements. Third, we employed weighted quantile sum (WQS) regression and Bayesian Kernel Machine Regression (BKMR) to evaluate the combined associations of trace element mixtures and to identify potential interactions among elements. Finally, we applied restricted cubic spline (RCS) models to explore dose–response relationships between individual trace elements and T2DM. By integrating multiple analytical methods, this study sought to provide a more comprehensive understanding of the associations between individual and combined trace element levels and T2DM.

## 2. Materials and Methods

This study was performed in accordance with the principles of the Declaration of Helsinki. The protocol was approved by the Ethics Committee of Ningxia Medical University (ethics approval No. 2018-021). All participants provided written informed consent prior to enrollment.

### 2.1. Study Population

This study was a 1:1 propensity score-matched case–control study based on age, sex, and residence, using baseline data from the Northwest China Natural Population Cohort: Ningxia Project (CNC-NX). Participants were selected via multistage cluster sampling from four townships in Wuzhong and Shizuishan cities. Permanent residents aged 35–74 years who provided written informed consent were recruited. The baseline survey was conducted from March 2018 to May 2019. After excluding participants with missing trace element measurements or incomplete biochemical data and applying the pre-specified inclusion and exclusion criteria, 1274 eligible participants (637 controls and 637 T2DM cases) were included.

T2DM was defined based on fasting plasma glucose (FPG), according to the 1999 World Health Organization (WHO) criteria [11]. Neither oral glucose tolerance testing nor HbA1c measurement was performed in this study. The inclusion criteria were: (1) aged 35–74 years; (2) no prior history of T2DM or use of antidiabetic medications; and (3) provision of written informed consent. The exclusion criteria included the following: the use of trace elements or nutritional supplements within the past month; presence of acute infection, severe trauma, recent surgery, or malignant tumor within the past month; severe liver or kidney dysfunction (based on self-reported history of liver or kidney disease at enrollment); long-term use of medications affecting glucose or trace element metabolism; mental or cognitive disorders.

Propensity score matching was performed using R software (version 4.2.0). Variables included in the propensity score model were sex, age, and residence. Propensity scores were estimated using logistic regression with case–control status as the dependent variable. Nearest neighbor matching without replacement was applied with a caliper width of 0.05 standard deviations of the logit of the propensity score, at a 1:1 matching ratio. Covariate balance before and after matching was assessed using standardized mean differences (SMDs), with an absolute SMD < 0.1 considered acceptable. The detailed participant selection and matching process is illustrated in Supplementary Figure S1.

### 2.2. General Information and Physical Examination

Demographic and lifestyle data were collected via a face-to-face questionnaire. Height and weight were measured using a stadiometer and multi-frequency bioelectrical impedance analysis (InBody 370, InBody Co., Ltd., Seoul, Korea), respectively. Blood pressure was measured twice using an electronic sphygmomanometer (OMRON Corporation, Kyoto, Japan), and the mean of the two readings was used for analysis. Dietary intake data were also collected via the questionnaire; however, they were not included in the current analysis and will be addressed in a separate investigation.

### 2.3. Blood Sample Collection and Measurements

Fasting blood samples were collected, separated, aliquoted, stored, and transported to the laboratory of Ningxia Medical University for measurement. FPG was measured using an automated biochemical analyzer. Fasting serum insulin (FINS) was measured using a chemiluminescence immunoassay analyzer. The homeostasis model assessment (HOMA) was used to evaluate insulin resistance and pancreatic β-cell function. The HOMA-IR index was calculated as: [FINS (μIU/mL) × FPG (mmol/L)]/22.5. The HOMA-β index was calculated as: [20 × FINS (μIU/mL)]/[FPG (mmol/L)–3.5].

### 2.4. Measurement of Serum Trace Elements Concentrations

Based on the established roles of trace elements in glucose homeostasis, insulin resistance, and oxidative stress, as well as their common combined levels in the general environment, we selected 18 trace elements for measurement. Samples were digested using a microwave digestion system (CEM MARS 6, CEM Corporation, Matthews, NC, USA) after pretreatment with concentrated nitric acid and ultrapure water, and then acid-driven at 130 °C for 155 min using an acid-driving instrument (VB24 UP, Beijing LabTech Instruments Co., Ltd., Beijing, China). Serum trace element concentrations were determined using a quadrupole inductively coupled plasma-mass spectrometer (ICP-MS; Agilent 7800, Agilent Technologies International Japan, Ltd., Tokyo, Japan). A standard curve (r > 0.999) was generated for each assay, with quality control samples analyzed every 20 samples. Quality control procedures included the analysis of the certified reference material (CRM) ClinChek^®^ Serum Control Level II (No. 8881; RECIPE Chemicals + Instruments GmbH, Munich, Germany) after every 20 samples to monitor calibration stability. The intra- and inter-assay coefficients of variation (CVs) for serum trace elements were all <15%. The recoveries ranged from 75.2% to 118.5%, confirming the accuracy and precision of the analytical method. Limits of detection (LODs) ranged from 0.0014 μg/L for cobalt (Co) to 3.1946 μg/L for aluminum (Al). Concentrations below the LOD were replaced with LOD/2 [12]. The proportions of measurements below the LOD for each element are presented in Supplementary Table S1.

### 2.5. Statistical Analysis

Normally distributed continuous variables are mean ± SD; skewed variables are median (IQR); categorical variables are *n* (%). Group comparisons used *t*-test, Mann–Whitney U, or chi-square test. Trace element concentrations were log-transformed and divided into quartiles. Spearman correlation assessed correlations among elements, classified as weak (r ≤ 0.3), moderate (0.3 < r < 0.8), or strong (r ≥ 0.8). To screen for key trace elements associated with T2DM, we performed LASSO-penalized logistic regression using the glmnet package (version 4.1) with a binomial logit link. All numeric predictors were standardized (standardize = TRUE). Covariates [age, sex, smoking, alcohol drinking, body mass index (BMI), hypertension, education level, physical activity] were not penalized (penalty factor = 0), while the 18 trace elements received a penalty factor of 1. The optimal tuning parameter λ was selected by ten-fold cross-validation based on λ.min (minimum deviance). Logistic regression (Model 1: single element; Model 2: adjusted for age, sex, smoking, alcohol drinking, BMI, and hypertension; Model 3: further adjusted for education level and physical activity) assessed associations between trace elements and T2DM, with ORs and 95% CIs calculated for quartiles and continuous (log-transformed) variables. WQS regression was performed using the gWQS package. For WQS regression, the 14 LASSO-selected trace elements were included as mixture components and categorized into quartiles. Both positive-direction (b1_pos = TRUE) and negative-direction (b1_pos = FALSE) models were fitted, with 100 bootstrap iterations and a 60:40 random split into training and validation sets; the final model was selected based on the lower AIC value. All covariates were included as adjustment variables. BKMR was performed using the bkmr package (version 0.2.0) with default priors: σ^2^ ~ Inv-Gamma(0.001, 0.001); ρ_m = 1/r_m, r_m ~ Uniform(0, 100); λ ~ Gamma(10, 10); π ~ Beta(2, 6). All trace element variables were centered and scaled. Convergence was assessed using four MCMC chains (10,000 iterations, 5000 warm-up) via bkmrhat, with R-hat < 1.01 and effective sample sizes > 400. Variable selection was enabled (varsel = TRUE), with importance quantified by PIPs. For binary T2DM, we specified family = ‘binomial’ (probit link) and set est.h = TRUE. To formally quantify pairwise interactions in BKMR, we computed contrast estimates by comparing the exposure–response function for one element when a second element was fixed at different quantiles (e.g., 25th, 50th, and 75th percentiles). Interaction was considered present if the 95% CI of the contrast did not cross zero. RCS (four knots at 5th, 35th, 65th, 95th percentiles) explored dose–response patterns. Analyses used SPSS 27.0 and R 4.5.2. All *p*-values were two-sided, with significance at *p* < 0.05.

## 3. Results

### 3.1. Baseline Characteristics of the Study Participants

Baseline characteristics are shown in Table 1. The two groups were well matched in terms of age and sex (*p* > 0.05). T2DM participants had higher BMI, hypertension, FPG, FINS, HOMA-IR, and proportions of smokers and alcohol drinkers, while exhibiting lower education level, lower physical activity level, and HOMA-β cell function index (all *p* < 0.001).

### 3.2. Trace Element Concentrations and Correlations

As shown in Table 2, serum levels of lithium(Li), Al, Zn, Se, strontium(Sr), and tellurium(Te) were significantly higher in the T2DM group than in the control group, while levels of boron(B) and lead(Pb) were significantly lower (all *p* < 0.05).

**Figure 1:**
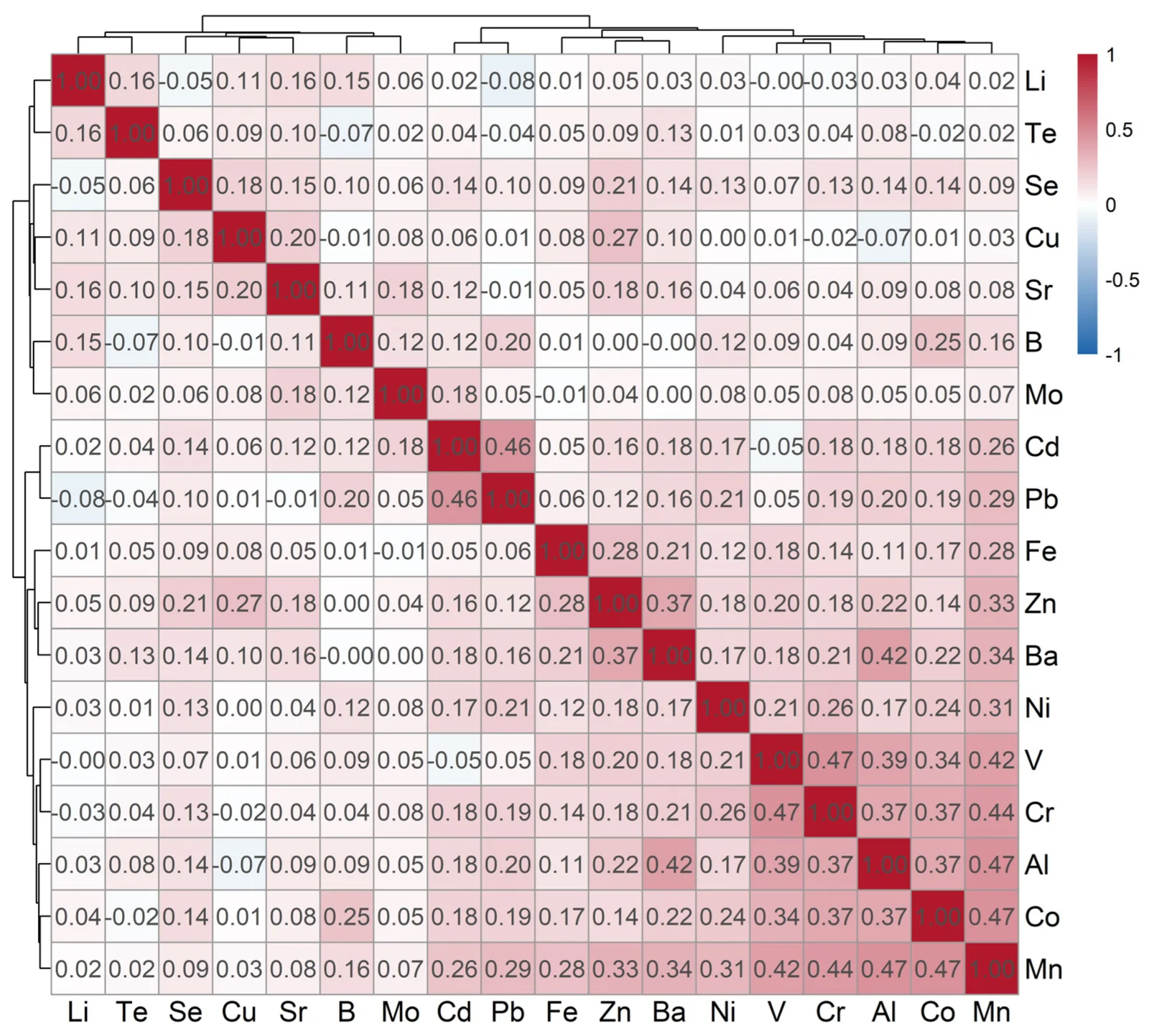
Correlation heatmap of 18 serum trace elements. Note: The heatmap displays the Spearman correlation coefficient matrix of 18 serum trace elements in the T2DM group. The color gradient represents the direction and magnitude of correlations, with red indicating positive correlations and blue indicating negative correlations; darker shades indicate stronger correlations (coefficients closer to 1 or −1).

Spearman rank correlation coefficients among log-transformed trace element concentrations in the T2DM group are shown in Figure 1. Positive correlations were observed for Co–Mn (0.47), Al–Mn (0.47), cadmium(Cd)–Pb (0.46), Mn–chromium(Cr) (0.44), V–Mn (0.42), V–Cr (0.47), and Al–barium(Ba) (0.42) (|r| > 0.3). Weak negative correlations (|r| < 0.3) were found for Li–Pb (−0.08), B–Te (−0.07), and Al–Cu (−0.07).

### 3.3. Identification of Key Trace Elements Associated with T2DM Using LASSO Regression

**Figure 2:**
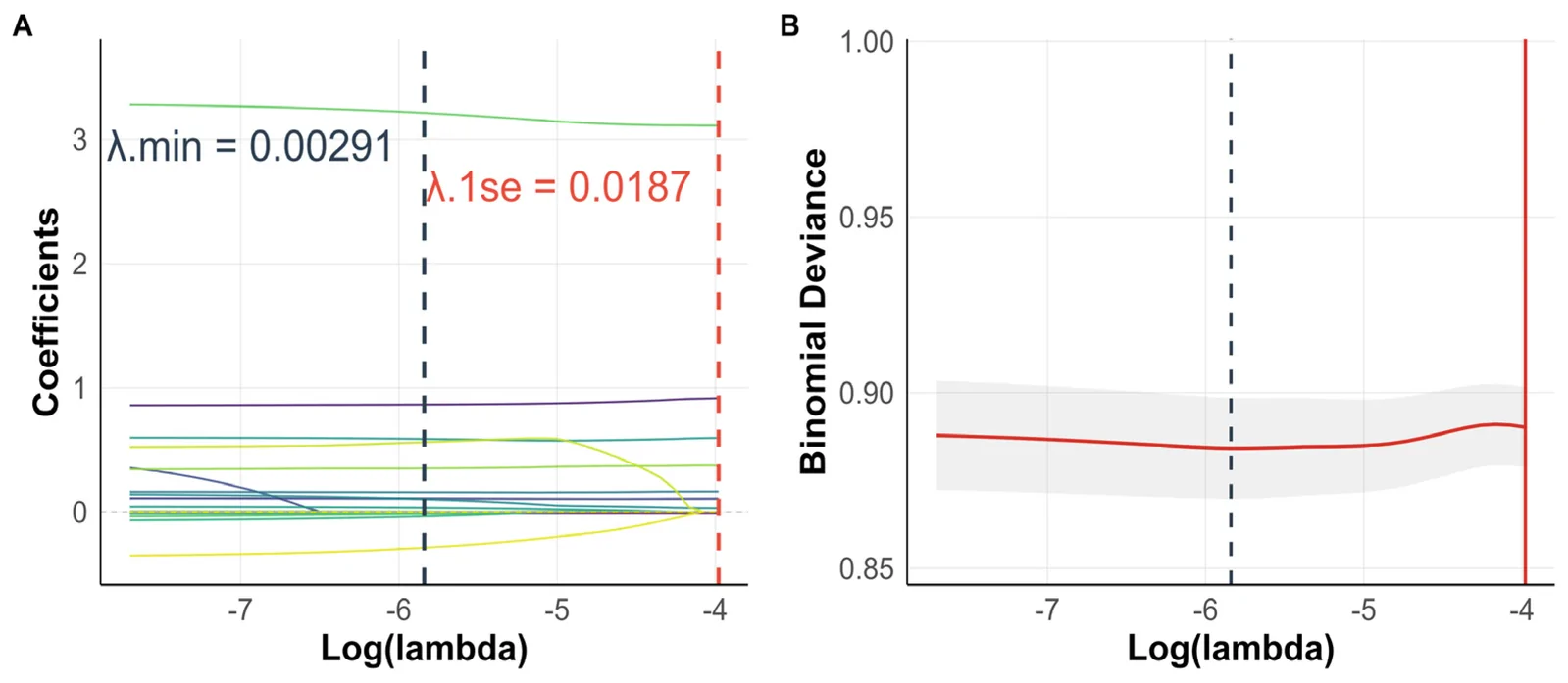
LASSO regression for the selection of key trace elements associated with T2DM. Note: In this study, age, sex, smoking, alcohol drinking, BMI, hypertension, education level, physical activity, and 18 trace elements were included in the LASSO regression model. The 8 covariates were assigned a penalty factor of 0 to be forced into the model without penalization, while the 18 trace elements received a penalty factor of 1 for LASSO variable selection. (**A**) Trajectories of regression coefficients for each trace element as a function of log(λ). Different colors represent different trace elements.The black dashed line indicates λ.min (λ = 0.00291), corresponding to the minimum cross-validated deviance, and the red dashed line indicates λ.1se (λ = 0.0187). (**B**) Cross-validated binomial deviance as a function of log(λ). Based on λ.min, 14 trace elements with non-zero coefficients were selected and included in the subsequent models.

LASSO regression (10-fold cross-validation; Figure 2) with the expanded covariate set (age, sex, smoking, alcohol drinking, BMI, hypertension, education level, physical activity) selected 14 trace elements with non-zero coefficients at λ.min = 0.0029: Li, V, Cu, Ba, B, Al, Cr, Mn, Ni, Se, Sr, molybdenum(Mo), Te, and Pb (see Table S2 for the correlation coefficients). No additional selection step was applied; the 14 LASSO-selected elements were included in subsequent models.

### 3.4. Associations Between Individual Trace Elements and T2DM Using Logistic Regression

Logistic regression was used to evaluate the associations between trace elements and T2DM (Table 3). In Model 1, Li, Al, Se, Sr, and Te were positively associated with T2DM, while B and Pb were negatively associated (all *p* < 0.05). Compared with the lowest quartile (Q1), the highest quartile (Q4) of Li, Al, Sr, Te, Ba, and Cu showed significantly elevated odds, whereas the Q4 groups of B and Pb showed significantly reduced odds. In Model 2, Li remained positively associated, and B and Pb remained negatively associated. Quartile analyses further revealed that, compared with Q1, the Q4 group of Li had significantly elevated odds, while the Q4 groups of B, Pb showed significantly reduced odds, and Ni showed a tendency toward reduced odds. In Model 3, Li and Al were positively associated, while B and V were negatively associated. Quartile analyses further demonstrated that, compared with Q1, the Q4 groups of Li, and Te had significantly elevated odds, Al showed a tendency toward increased odds. whereas the Q4 groups of B, V, and Ni showed significantly reduced odds.

A sensitivity analysis was performed by removing physical activity from Model 3 while retaining the other covariates (see Supplementary Table S3). The results showed that V remained inversely associated with T2DM (OR = 0.73, 95% CI: 0.57, 0.97, *p* = 0.007).

Multivariable linear regression models were used to evaluate the associations between trace elements and FPG as a continuous outcome (see Supplementary Table S4). In Model 1, Li and Cu were positively associated with FPG, while B and Pb were negatively associated (all *p* < 0.05). Compared with Q1, the Q4 of Li and Cu were associated with significantly higher FPG levels, whereas the Q4 groups of B and Pb showed significantly lower FPG levels. In Model 2, Li and Cu remained positively associated, while B, V, and Pb were negatively associated. Quartile analyses further revealed that, compared with Q1, the Q4 groups of Li and Cu were associated with significantly higher FPG levels.

### 3.5. WQS Regression for the Association Between Trace Elements and T2DM

**Figure 3:**
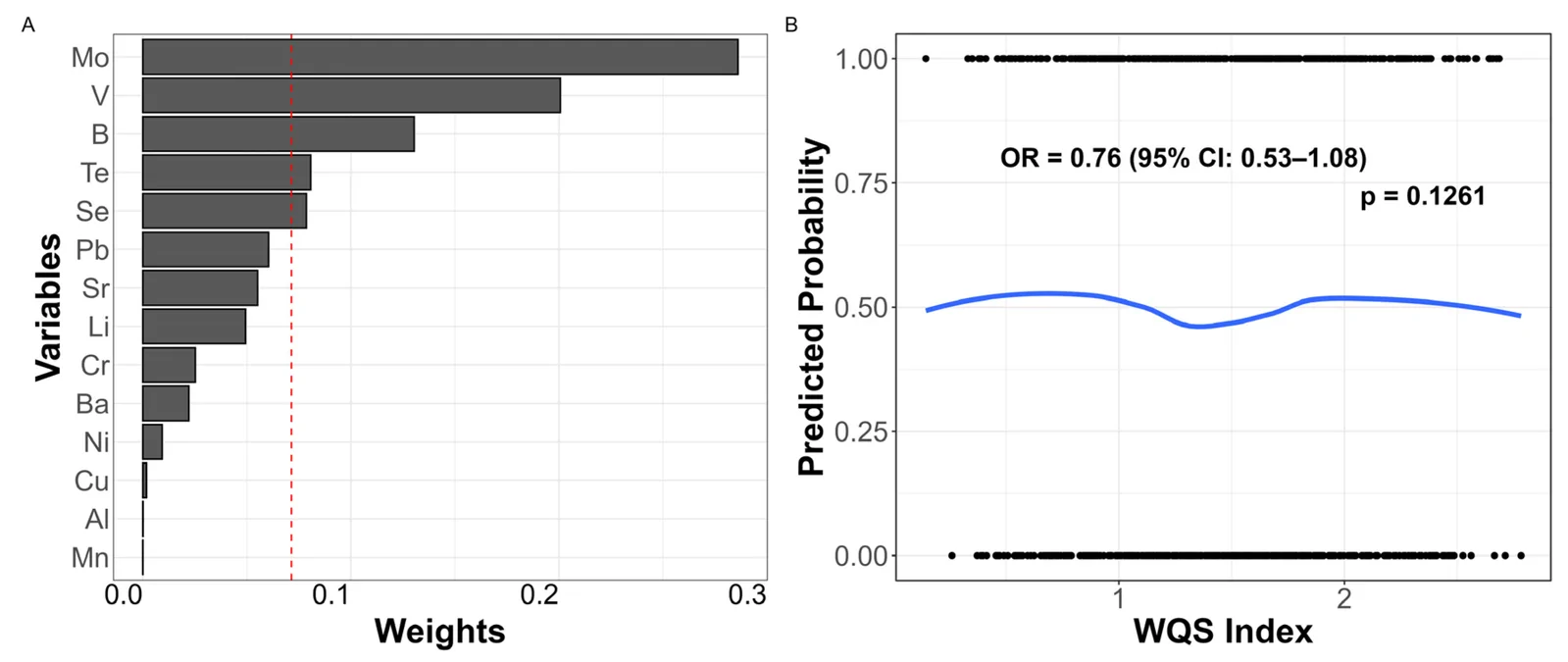
WQS regression for the association between trace element mixtures and T2DM. Note: Results of weighted quantile sum (WQS) regression for the association between the 14-trace-element mixture and T2DM. (**A**) Bar chart displaying the mean weights of each serum trace element in the WQS regression model, reflecting their relative contributions to the joint association. The red dashed line is a reference line representing the average weight (approximately 1/14 ≈ 0.07), used to distinguish which trace elements contribute more than the average to the overall mixture effect. (**B**) Fitted line showing the relationship between the WQS index and the predicted probability of T2DM, along with the 95% CI. The WQS index was constructed using the 14 trace elements divided into quartiles (q = 4). Per one-quartile increment in the WQS index, the estimated OR was 0.7555 (95% CI: 0.53–1.08, *p* = 0.126), adjusted for age, sex, smoking, alcohol drinking, BMI, hypertension, education level, and physical activity.

WQS regression was used to evaluate the joint associations between the mixture of trace elements and T2DM. Both positive and negative direction models were fitted separately, and the better model was selected based on the AIC value; the negative direction model fit better (lower AIC) and was therefore reported as the final result. As shown in Figure 3, Mo (0.286), V (0.201), and B (0.130) were the predominant contributors to the mixture, with the remaining elements exhibiting progressively lower weights (see Supplementary Table S5 for weights). The WQS index yielded a regression coefficient of β = −0.2804, corresponding to an OR of 0.7555 (95% CI: 0.53–1.08, *p* = 0.126) (Figure 3).

### 3.6. BKMR Model for the Association Between Trace Element Mixtures and T2DM

**Figure 4:**
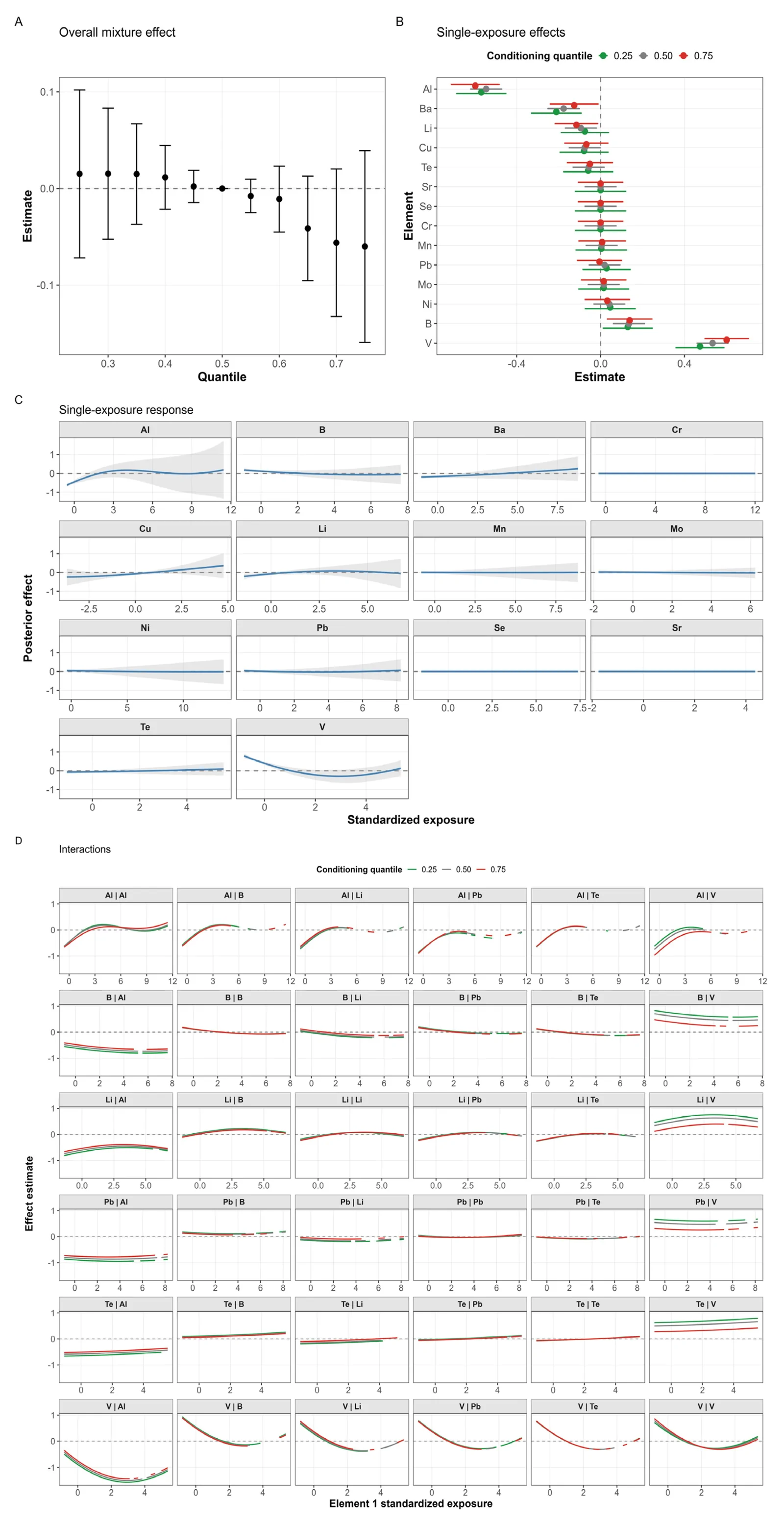
BKMR model for the individual and overall associations and interactions of trace element mixtures with T2DM. (**A**) Overall mixture-effect plot. The x-axis represents quantiles of the mixed-exposure index; the y-axis is the effect estimate. The horizontal dashed line at an estimate of 0 indicates no association. Vertical bars denote 95% CIs. (**B**) Single-element conditional estimates for each trace element when the remaining trace elements were fixed at the 25th, 50th, and 75th quantiles. The x-axis is the effect estimate; the vertical dashed line at an estimate of 0 means no effect. (**C**) Single-exposure-response curves. Each sub-plot shows the posterior estimate of an individual trace element, while all other trace elements were held constant at their median concentration. The x-axis represents standardized concentration, and the y-axis denotes the posterior estimate. The gray shaded area corresponds to the 95% CI. (**D**) Two-way interaction plots. Each sub-graph illustrates the exposure-response curve of the first element, whereas the paired second element is fixed at its 25th, 50th, and 75th quantiles, and the remaining trace elements are held at median values. The x-axis represents standardized concentration of the first element, and the y-axis is the posterior association estimate. The gray dashed line is the zero-effect reference line at Y = 0, used to determine whether the effect estimate is positive, negative, or indistinguishable from no association; curves above the dashed line indicate a positive association trend, curves below it indicate a negative association trend, and curves crossing the dashed line suggest non-significance. Separation among the three curves suggests potential pairwise interaction.

BKMR was used to evaluate the overall joint association of the mixture of 14 serum trace elements with T2DM (Figure 4A). At all mixture quantiles, the 95% posterior CI crossed the null. This indicates that the overall mixture of the 14 trace elements was not significantly associated with T2DM.

The single-exposure point estimates (Figure 4B) and continuous concentration–response curves (Figure 4C) suggested possible nonlinear associations for some elements. For Al, the single-exposure point estimates were negative when other elements were fixed at the 25th, 50th, and 75th percentiles (Figure 4B), while the continuous concentration–response curve showed a tendency toward increased association with T2DM as Al concentration increased (Figure 4C). For V, the single-exposure point estimates were positive when other elements were fixed at the 25th, 50th, and 75th percentiles (Figure 4B), while the continuous concentration–response curve exhibited a U-shaped pattern—protective at low levels and increased association at high levels (Figure 4C).

Figure 4D presents the pairwise interaction effects among all trace elements. For V, when the paired elements were fixed at different percentiles, the effect curves showed some separation, suggesting possible effect modification by Al, B, Li, Pb, and Te. However, formal interaction contrasts showed that all 95% posterior intervals crossed zero, indicating no statistically significant interactions (Supplementary Table S6). The remaining element pairs largely overlapped, suggesting no apparent interactions. Overall, these curve separations represent exploratory point-estimate signals rather than statistically significant interactions.

### 3.7. RCS Models for Visualizing the Associations Between Trace Elements and T2DM

**Figure 5:**
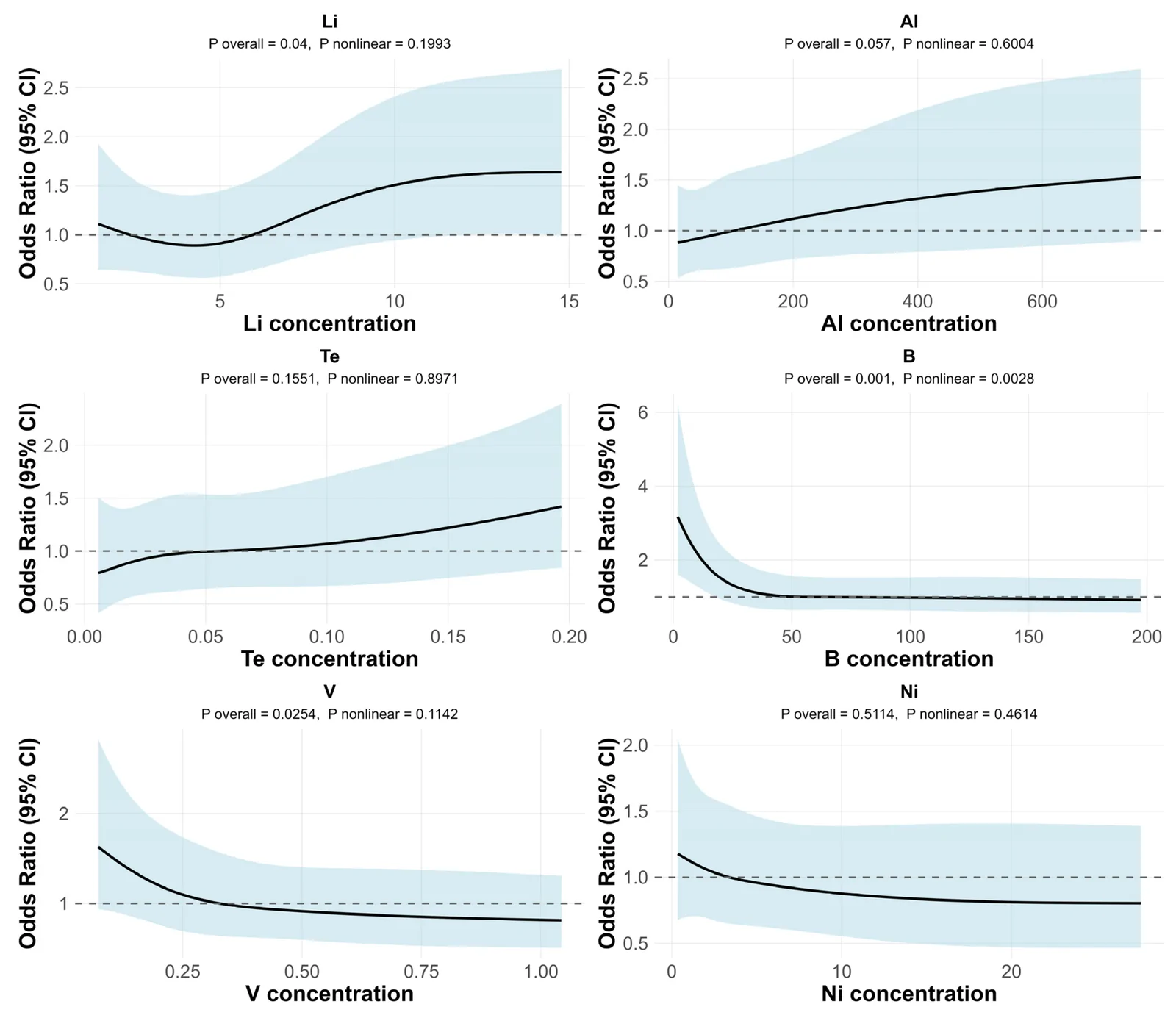
RCS models for the dose–response relationships between selected trace elements and T2DM. Note: Adjusted for age, sex, smoking, alcohol drinking, BMI, hypertension, education level, and physical activity. The black solid curves represent the ORs, and blue shaded areas indicate the corresponding 95% CIs. The horizontal dashed line at OR = 1 indicates no association. The six panels, arranged from left to right and top to bottom, show the dose–response relationships between serum concentrations of Li, Al, Te, B, V, and Ni and T2DM, respectively. P-overall denotes the *p*-value for the overall association, and P-nonlinear represents the *p*-value for the nonlinear test.

The shape of the dose–response curves observed in the RCS analysis should be interpreted as descriptive and exploratory. These curves do not represent formally tested thresholds or plateaus, and any patterns suggested by the curves require further validation in future studies. RCS models showed that serum B was significantly associated with T2DM overall (P-overall = 0.001), and exhibited a significant non-linear dose–response relationship (P-nonlinear = 0.0028). In the low-concentration range, the OR for T2DM decreased rapidly with increasing serum B concentrations; after which the curve tended to level off. Serum V and Li both showed significant overall associations with T2DM (P-overall = 0.0254 and 0.0400, respectively), but the tests for non-linearity were not statistically significant (P-nonlinear = 0.1142 and 0.1993, respectively), suggesting no non-linear dose–response relationship for either element.As for serum Al, Te, and Ni, there was no overall association with T2DM, and the tests for non-linearity were not statistically significant; thus, no non-linear dose–response relationship was supported for these elements (Figure 5).

## 4. Discussion

In this 1:1 propensity score-matched case–control study, we employed a combination of multiple statistical methods—including LASSO regression, logistic regression, WQS, BKMR, and RCS—to systematically evaluate the associations between 18 serum trace elements, at both individual and combined levels, and T2DM. Overall, Li and Te showed positive associations across multiple analyses, whereas B, V, and Pb exhibited inverse associations across multiple analytical methods. However, the strength of evidence varied across elements; the association for Li was the most robust, while the evidence for Te was less consistent; the association for Al should be interpreted with caution because of possible pre-analytical contamination. WQS showed a negative but non-significant trend for the mixed exposure index, and BKMR did not identify a statistically significant overall association between the mixture and T2DM. Although the BKMR bivariate interaction plots suggested some separation in point estimates for the effect curves of V with Al, B, Li, Pb, and Te, all 95% posterior intervals for the formal interaction contrasts crossed zero and did not reach statistical significance. Therefore, these results can only be considered potential signals of effect modification rather than confirmed interactions. RCS revealed a significant non-linear dose–response pattern for B (P-nonlinear = 0.0028), whereas Li and V showed linear associations (positive for Li and negative for V).

These observations, however, should be interpreted with caution, as the 18 elements are not biologically homogeneous. Based on their physiological functions, they can be broadly classified into essential trace elements [Zn, Se, Cu, Mn, Mo, Cr, Ni, Fe(iron)], potentially beneficial elements (Li, B, Sr, V, Te), and toxic heavy metals [Pb, Cd, Arsenic(As), Al, antimony (Sb)]. These categories differ fundamentally in their primary sources (dietary intake vs. environmental exposure), metabolic regulation (active homeostatic control vs. passive accumulation), and biological effects (physiological necessity vs. potential toxicity) [13]. Therefore, their associations with T2DM should be interpreted separately by category, rather than treated as a unified ‘trace element’ construct.

It is important to acknowledge that single serum measurements of trace elements have inherent limitations as biomarkers of long-term environmental exposure, as the temporal windows reflected by serum concentrations vary substantially across elements due to differences in biological half-lives. For essential trace elements such as Se and Zn, serum levels are predominantly influenced by recent dietary intake, reflecting short-term physiological status over days to weeks [14]. For Cu, serum concentrations are subject to complex homeostatic regulation and acute-phase responses, making their interpretation as exposure indicators particularly challenging [15]. For toxic metals such as Cd and Pb, it is critical to distinguish between serum concentration and body burden. Although Cd has a biological half-life of 10–30 years in the kidney and other storage compartments, and bone Pb has a half-life of approximately 25–30 years, their serum concentrations primarily reflect the labile pool recently mobilized from tissues or recent external uptake, rather than the cumulative long-term dose [16]. Blood Cd has been reported to have a half-life of weeks to months, while plasma Pb has a biological half-time of approximately one month [17]. Urinary Cd and bone Pb are recognized as superior biomarkers for assessing chronic cumulative exposure [18]. Therefore, the associations observed in the present study should be interpreted as correlations between serum trace element status and T2DM, rather than causal effects of long-term environmental exposure.

Li and Te were positively associated with T2DM across multiple statistical methods, although the strength of evidence differed between the two elements. The evidence for Li was more robust, whereas the evidence for Te was limited and inconsistent. Li has been previously reported to be positively associated with T2DM and insulin resistance in several cross-sectional and case–control studies, which is consistent with our findings [19]. Mechanistically, Li inhibits GSK-3β, a negative regulator of insulin signaling, and chronic Li exposure may promote IRS-1 phosphorylation and insulin resistance [20]. Li also interferes with thyroid hormone synthesis, and Li-induced subclinical hypothyroidism has been linked to increased T2DM risk [21]. However, the evidence for Te was less consistent. In logistic regression Model 3, the continuous association for Te was not statistically significant (OR = 0.170, 95% CI: 0.989–1.385). In contrast, quartile analysis showed a significant positive association in the highest quartile (Q4 vs. Q1) and a significant trend across quartiles (*p* for trend = 0.0283), suggesting a possible non-linear association that may not be captured by a single linear term. Therefore, the association between Te and T2DM should be interpreted with caution and requires further validation. Despite these inconsistent findings, Te has received limited epidemiological attention. The few available studies have reported positive associations between serum Te levels and elevated blood glucose, oxidative stress, and inflammation, broadly consistent with our findings [22]. Mechanistically, Te may induce oxidative stress and, as a sulfur analog, competitively inhibit selenoenzymes such as glutathione peroxidase, thereby compromising antioxidant defense and exacerbating insulin resistance [23].

B, V, and Pb consistently demonstrated inverse associations with T2DM across multiple analytical methods. B was among the most robust negative correlates of T2DM, identified by LASSO as a negative predictor and retaining significant negative associations in logistic regression Models 1, 2, and 3. Epidemiological evidence on B and T2DM remains inconsistent: one HUNT3 study reported a positive association [24], while another study found no significant association [25], possibly due to differences in study design and population characteristics. The inverse association between serum B and T2DM should be interpreted with caution. Serum B concentrations are derived primarily from plant-based foods (e.g., fruits, vegetables, legumes, and nuts), and dietary patterns rich in these foods have been consistently associated with lower T2DM risk [26]. Because detailed dietary intake data were not included as covariates in our analysis, we cannot distinguish whether the observed inverse association reflects a biological effect of B itself or is attributable to the protective effects of an overall healthy dietary pattern. Although education level—a proxy for socioeconomic status and a strong predictor of dietary quality [27]—was adjusted for in our models and may partially control for population-level dietary pattern confounding, it does not fully capture individual-level dietary intake. Thus, residual confounding by diet cannot be excluded. This finding should be regarded as hypothesis-generating and requires validation in future prospective studies incorporating detailed dietary assessments, such as food frequency questionnaires or dietary pattern scores (e.g., Healthy Eating Index, Alternative Healthy Eating Index), to further disentangle the independent contributions of B itself and overall dietary patterns to T2DM.

V was identified as a key variable by LASSO regression. In logistic regression, V was significantly inversely associated with T2DM only in Model 3 (which included education level and physical activity), but not in Models 1 or 2. In the WQSanalysis, V ranked second in WQS weight (0.201), indicating a substantial contribution to the joint association. BKMR single-element conditional effect estimates showed that the point estimates for the effect of V varied to some extent when the remaining elements were fixed at different quantiles (25th, 50th, and 75th percentiles); the BKMR continuous concentration–response curve suggested a U-shaped pattern for V—point estimates tending toward protection at low concentrations and toward increased association at high concentrations—although the 95% CIs all crossed the null value and did not reach statistical significance. The bivariate interaction plots further showed that, when the paired elements were fixed at different quantiles, the effect curves of V with Al, B, Li, Pb, and Te exhibited some degree of separation or shift in point estimates, suggesting possible effect modification; however, the formal interaction contrasts showed that all 95% posterior intervals for V with the above elements crossed zero and did not reach statistical significance. These interaction signals should be cautiously interpreted as exploratory and require further validation in larger prospective studies. RCS analysis showed a negative linear association between V and T2DM. The inverse association observed in logistic regression, together with the non-linear U-shaped pattern revealed by BKMR, may reflect the complex and concentration-dependent biological effects of V. Previous epidemiological studies have reported inverse associations between Vand T2DM [28,29], consistent with our findings, and animal studies have demonstrated that V compounds exert insulin-mimetic and insulin-sensitizing effects, improving glycemic control [30]. Mechanistically, vanadate and peroxovanadium compounds can inhibit protein tyrosine phosphatases, thereby enhancing insulin receptor kinase activity, activating the PI3K/Akt signaling pathway, promoting GLUT4 translocation, and facilitating glucose uptake [31]. However, high-dose V exposure may induce oxidative stress, mitochondrial dysfunction, and β-cell damage, which could explain the U-shaped pattern observed in BKMR [32].

Pb consistently demonstrated inverse associations with T2DM across LASSO regression and logistic regression models 1 and 2. Given the cross-sectional design of this study, causal inference cannot be made, and the inverse association between serum Pb and T2DM should be interpreted with caution. Multiple non-causal explanations may account for this finding, including reverse causation (diabetes-related metabolic or renal changes affecting Pb levels) [33,34], residual confounding (socioeconomic, lifestyle, or dietary factors) [35], exposure measurement limitations (serum Pb reflects recent exposure rather than long-term body burden; blood Pb has a half-life of approximately 30–40 days, whereas bone Pb reflects cumulative exposure over decades) [36], and potential selection bias. While Recent studies have similarly reported inverse associations between Pb and T2DM in some populations [37]; however, the interpretation of these findings remains controversial. The relationship between Pb and T2DM may be non-linear (e.g., U-shaped or L-shaped), with the direction of association potentially differing across exposure levels. Although the inverse association observed in this study was statistically significant, we emphasize that it should not be interpreted as evidence of a protective effect of Pb. This observation should therefore be regarded as hypothesis-generating and requires confirmation in future prospective cohort studies, Mendelian randomization analyses, or experimental investigations.

BKMR was used to evaluate the joint associations of the trace element mixture with T2DM and potential interactions among trace elements. The overall concentration–response results showed no statistically significant association between the trace element mixture and T2DM, and the 95% posterior CIs crossed the null at all mixture quantile levels. No significant pairwise interactions were identified. It should be noted that the LASSO, WQS, and BKMR analyses were performed on the same study population, and thus their convergence does not constitute independent external replication. Nevertheless, these methods are mathematically and algorithmically distinct—LASSO is a penalized regression with L1 shrinkage for variable selection, WQS is a weighted index approach that summarizes mixture components into a single score, and BKMR is a non-parametric Bayesian kernel regression that flexibly models non-linear relationships and interactions. The consistency of findings across these three methodologically diverse approaches supports the internal robustness of our results.

Several limitations of this study should be acknowledged. First, the cross-sectional design precludes any inference regarding causality or temporality, and reverse causation cannot be ruled out, as diabetes-related metabolic disturbances—including osmotic diuresis, chronic inflammation, and dietary changes that may occur even before clinical diagnosis—may alter circulating trace element concentrations. In addition, trace element measurements were based on a single serum sample, which may not fully capture long-term exposure status due to variations in biological half-lives across different elements and potential within-individual variability over time. The directionality of these associations remains uncertain, and future prospective cohort studies or Mendelian randomization analyses are needed to clarify the temporal and causal nature of the relationship between circulating trace elements and T2DM. Second, the serum Al concentrations in this study were markedly higher than the general population reference values, likely due to Al released from collection materials. Therefore, the Al results should be interpreted with caution and considered exploratory. Third, although we adjusted for a comprehensive set of established confounders, we cannot fully exclude the possibility of residual confounding, particularly for the inverse associations observed for B and Pb, given their complex relationships with dietary and lifestyle factors. In addition, detailed dietary intake data were not included in the current analysis, and we therefore cannot rule out the possibility that differences in dietary intake may account for some of the observed associations. Fourth, detailed renal function parameters (e.g., serum creatinine, eGFR), HbA1c, and disease duration records werenot available in this study; although participants with self-reported severe kidney disease were excluded, residual variation in renal function within the included population may still have influenced trace element concentrations. Moreover, diabetes was defined based solely on fasting plasma glucose, without oral glucose tolerance testing or HbA1c measurement, which may underestimate the true prevalence of diabetes and limits our ability to assess associations with longer-term glycemic control or disease progression.Fifth, the three analytical approaches (LASSO, WQS, and BKMR) were applied to the same dataset, and thus their convergence does not constitute independent replication; rather, it reflects consistency across methodologically distinct models. Finally, our study population was derived from a specific region in Northwest China, which may limit the generalizability of our findings to other populations with different dietary patterns, environmental exposures, or ethnic backgrounds. Future prospective cohort studies and external validations in independent populations are warranted to confirm our findings.

## 5. Conclusions

In this 1:1 propensity score-matched case–control study, we applied a sequential analytical strategy—including LASSO, logistic regression, WQS, BKMR, and RCS—to evaluate the associations of 18 serum trace elements with T2DM. After LASSO selection, 14 elements were included in subsequent models. We found that Li and Te were positively associated with T2DM, whereas B, Pb, and V showed inverse associations across multiple methods; however, the evidence for Te was less consistent, with a non-significant continuous association but a significant highest quartile and trend. WQS showed a negative but non-significant trend for the mixture index, with V and B as the main contributors. BKMR did not identify a statistically significant overall association or significant pairwise interactions; although point estimates suggested possible effect modification involving V with Al, B, Li, Pb, and Te, formal interaction contrasts were not statistically significant. RCS revealed a significant non-linear dose–response pattern for B (P-nonlinear = 0.0028), while Li and V showed linear associations (positive for Li, negative for V). These findings should be considered exploratory and require validation in prospective studies.

## Figures and Tables

**Table 1:** Characteristics of the control and T2DM groups.

Variable	Control (*n* = 637)	T2DM (*n* = 637)	*p*
Age	60.32 ± 9.40	60.83 ± 9.39	0.328
Sex, *N* (%)			0.117
Male	222 (34.9%)	250 (39.2%)	
Female	415 (65.1%)	387 (60.8%)	
Smoking			<0.001
N	602 (94.5%)	557 (87.4%)	
Y	35 (5.5%)	80 (12.6%)	
Alcohol Drinking			<0.001
N	578 (90.7%)	490 (76.9%)	
Y	59 (9.3%)	147 (23.1%)	
Education level			<0.001
Middle school or below	307 (48.2%)	362 (56.8%)	
High school or above	330 (51.8%)	275 (43.2%)	
Physical activity			<0.001
low	591 (92.8%)	207 (32.5%)	
moderate	46 (7.2%)	306 (48.0%)	
high	0 (0.0)	124 (19.5%)	
BMI (kg/m^2^)	24.71 ± 3.33	25.83 ± 3.49	<0.001
Hypertension (Yes, %)	169 (26.5%)	233 (36.6%)	<0.001
FPG (mmol/L)	5.30 (4.95, 5.67)	8.45 (7.48, 11.43)	<0.001
FINS (μIU/mL)	6.08 (4.48, 8.91)	7.62 (5.22, 10.89)	<0.001
HOMA-IR	1.43 (0.99, 2.05)	3.17 (2.13, 4.75)	<0.001
HOMA-β	71.64 (47.80, 101.26)	28.04 (15.55, 45.13)	<0.001

Note: Values are means ± SDs for normally distributed data, medians (IQRs) for non-normally distributed data, or *n* (%) for categorical data. Comparisons between case and control groups were performed with Student’s *t*-test or Mann–Whitney U test for continuous variables, and chi-square test for categorical variables.

**Table 2:** Comparison of serum trace element concentrations between the control and T2DM groups.

Variable	Control (*n* = 637)	T2DM (*n* = 637)	*p*
Li	5.64 (3.75, 8.23)	6.24 (4.02, 9.95)	0.0038
B	60.66 (40.52, 100.32)	54.17 (30.92, 86.09)	<0.001
Cr	1.95 (0.91, 4.18)	2.00 (0.92, 4.82)	0.5884
Al	91.66 (44.7, 202.05)	112.48 (47.39, 266.13)	0.0068
V	0.31 (0.19, 0.56)	0.35 (0.19, 0.59)	0.3166
Mn	2.44 (1.34, 4.84)	2.75 (1.49, 5.11)	0.1376
Fe	1325.11 (1015.37, 1694.72)	1363.61 (1019.28, 1754.75)	0.3699
Co	0.13 (0.09, 0.24)	0.13 (0.08, 0.21)	0.0936
Cu	1022.47 (889.73, 1153.76)	1040.26 (909.19, 1195.88)	0.0799
Zn	778.75 (626.33, 942.84)	811.21 (662.19, 1003.39)	0.0137
Se	92.86 (80.00, 107.49)	94.90 (81.06, 114.07)	0.0094
Sr	92.63 (76.60, 110.56)	97.14 (78.88, 118.84)	0.0045
Mo	1.21 (0.94, 1.51)	1.23 (0.90, 1.58)	0.8926
Cd	0.05 (0.03, 0.09)	0.05 (0.03, 0.08)	0.4430
Te	0.05 (0.03, 0.08)	0.06 (0.03, 0.10)	0.0209
Ni	3.77 (1.75, 7.38)	3.23 (1.65, 6.46)	0.2046
Ba	20.11 (13.88, 30.53)	21.19 (14.47, 43.40)	0.0617
Pb	1.19 (0.51, 2.20)	0.98 (0.36, 2.09)	0.0041

Note: all trace elements are presented as median (interquartile range, IQR) in μg/L.

**Table 3:** Associations of 14 trace elements with T2DM assessed by logistic regression.

Variable	Continuous	Q1	Q2	Q3	Q4	*p* for Trend
	OR (95% CI)		OR (95% CI)	OR (95% CI)	OR (95% CI)	
Li						
Model 1	1.196 (1.022, 1.400) *	1.00	1.085 (0.795, 1.481)	0.921 (0.674, 1.257)	1.703 (1.244, 2.330) *	0.0047
Model 2	1.216 (1.031, 1.434) *	1.00	1.125 (0.813, 1.556)	0.924 (0.667, 1.279)	1.822 (1.313, 2.528) *	0.0024
Model 3	1.268 (1.016, 1.584) *	1.00	1.141 (0.750, 1.737)	0.722 (0.469, 1.111)	1.901 (1.253, 2.886) *	0.0240
B						
Model 1	0.780 (0.701, 0.868) *	1.00	0.493 (0.359, 0.677) *	0.567 (0.413, 0.777) *	0.503 (0.367, 0.690) *	0.0002
Model 2	0.778 (0.697, 0.869) *	1.00	0.457 (0.329, 0.636) *	0.542 (0.391, 0.752) *	0.511 (0.368, 0.709) *	0.0004
Model 3	0.770 (0.669, 0.887) *	1.00	0.553 (0.364, 0.840) *	0.563 (0.370, 0.858) *	0.566 (0.373, 0.860) *	0.0144
Al						
Model 1	1.120 (1.023, 1.225) *	1.00	0.944 (0.693, 1.289)	1.230 (0.902, 1.679)	1.387 (1.016, 1.893) *	0.0133
Model 2	1.091 (0.993, 1.225)	1.00	0.899 (0.651, 1.283)	1.271 (0.921, 1.755)	1.243 (0.898, 1.721)	0.0565
Model 3	1.157 (1.024, 1.306) *	1.00	0.952 (0.626, 1.449)	1.396 (0.921, 2.117)	1.397 (0.921, 2.119)	0.0396
Se						
Model 1	1.680 (1.154, 2.447) *	1.00	1.006 (0.737, 1.373)	1.019 (0.747, 1.390)	1.352 (0.991, 1.847)	0.0677
Model 2	1.465 (0.990, 2.447)	1.00	0.961 (0.697, 1.325)	0.893 (0.646, 1.235)	1.250 (0.903, 1.730)	0.2577
Model 3	1.581 (0.963, 2.596)	1.00	1.049 (0.699, 1.572)	0.790 (0.519, 1.202)	1.202 (0.792, 1.823)	0.7148
Sr						
Model 1	1.684 (1.149, 2.467) *	1.00	0.875 (0.641, 1.196)	1.127 (0.826, 1.537)	1.405 (1.029, 1.919) *	0.0115
Model 2	1.404 (0.937, 2.467)	1.00	0.842 (0.610, 1.162)	0.940 (0.678, 1.303)	1.236 (0.890, 1.715)	0.1603
Model 3	0.928 (0.550, 1.566)	1.00	0.725 (0.477, 1.101)	0.929 (0.615, 1.403)	0.837 (0.549, 1.277)	0.6666
V						
Model 1	1.024 (0.903, 1.162)	1.00	0.876 (0.642, 1.196)	1.085 (0.795, 1.481)	1.078 (0.790, 1.471)	0.3808
Model 2	1.052 (0.921, 1.203)	1.00	0.932 (0.674, 1.289)	1.210 (0.873, 1.678)	1.140 (0.820, 1.585)	0.2213
Model 3	0.763 (0.642, 0.906) *	1.00	0.665 (0.444, 0.997) *	0.715 (0.471, 1.086)	0.498 (0.323, 0.767) *	0.0036
Te						
Model 1	1.133 (1.001, 1.282) *	1.00	0.994 (0.728, 1.356)	0.945 (0.692, 1.289)	1.479 (1.082, 2.021) *	0.0260
Model 2	1.102 (0.969, 1.254)	1.00	1.008 (0.730, 1.392)	0.935 (0.678, 1.292)	1.337 (0.966, 1.851)	0.1326
Model 3	0.170 (0.989, 1.385)	1.00	1.210 (0.791, 1.852)	1.166 (0.765, 1.777)	1.660 (1.090, 2.528) *	0.0283
Pb						
Model 1	0.858 (0.784, 0.939) *	1.00	0.753 (0.551, 1.029)	0.716 (0.524, 0.978) *	0.694 (0.508, 0.948) *	0.0228
Model 2	0.832 (0.757, 0.915) *	1.00	0.821 (0.594, 1.136)	0.717 (0.519, 0.992) *	0.652 (0.471, 0.904) *	0.0071
Model 3	0.895 (0.794, 1.009)	1.00	0.849 (0.559, 1.288)	0.727 (0.478, 1.108)	0.815 (0.539, 1.233)	0.2649
Ba						
Model 1	1.054 (0.939, 1.183)	1.00	1.012 (0.747, 1.390)	0.921 (0.675, 1.257)	1.479 (1.082, 2.021) *	0.0335
Model 2	0.976 (0.864, 1.103)	1.00	0.977 (0.708, 1.346)	0.800 (0.578, 1.106)	1.270 (0.914, 1.767)	0.3681
Model 3	1.075 (0.916, 1.262)	1.00	1.240 (0.814, 1.889)	0.889 (0.578, 1.369)	1.694 (1.109, 2.588) *	0.0657
Cr						
Model 1	1.031 (0.943, 1.128)	1.00	0.981 (0.719, 1.339)	0.945 (0.693, 1.289)	1.163 (0.852, 1.586)	0.4086
Model 2	1.023 (0.932, 1.123)	1.00	0.956 (0.692, 1.320)	0.983 (0.712, 1.356)	1.136 (0.823, 1.568)	0.4336
Model 3	0.978 (0.866, 1.103)	1.00	1.145 (0.756, 1.728)	1.080 (0.713, 1.636)	1.002 (0.658, 1.525)	0.9431
Cu						
Model 1	1.257 (0.840, 1.881)	1.00	1.113 (0.815, 1.519)	1.155 (0.847, 1.577)	1.387 (1.016, 1.893) *	0.0427
Model 2	1.192 (0.781, 1.817)	1.00	1.034 (0.747, 1.431)	1.103 (0.796, 1.531)	1.325 (0.954, 1.840)	0.0867
Model 3	1.054 (0.623, 1.782)	1.00	1.656 (1.084, 2.531) *	1.372 (0.887, 2.122)	1.647 (1.070, 2.535) *	0.0690
Mn						
Model 1	1.048 (0.940, 1.168)	1.00	0.921 (0.675, 1.257)	1.200 (0.879, 1.638)	1.192 (0.874, 1.626)	0.1148
Model 2	1.042 (0.930, 1.167)	1.00	0.918 (0.664, 1.268)	1.197 (0.866, 1.654)	1.183 (0.856, 1.633)	0.1405
Model 3	1.037 (0.894, 1.202)	1.00	0.953 (0.626, 1.451)	1.321 (0.872, 1.999)	0.142 (0.751, 1.736)	0.2775
Mo						
Model 1	0.938 (0.779, 1.129)	1.00	0.833 (0.611, 1.137)	0.823 (0.603, 1.123)	1.052(0.771, 1.435)	0.7830
Model 2	0.925 (0.761, 1.125)	1.00	0.835 (0.605, 1.152)	0.817 (0.591, 1.130)	1.023 (0.739, 1.421)	0.9356
Model 3	0.787 (0.612, 1.012)	1.00	0.672 (0.444, 1.016)	0.596 (0.394, 0.904) *	0.733 (0.482, 1.113)	0.1111
Ni						
Model 1	0.948 (0.872, 1.030)	1.00	1.100 (0.806, 1.501)	0.802 (0.588, 1.095)	0.818 (0.600, 1.116)	0.0677
Model 2	0.936 (0.858, 1.021)	1.00	1.077 (0.780, 1.487)	0.772 (0.558, 1.068)	0.762 (0.552, 1.053)	0.0283
Model 3	0.917 (0.820, 1.026)	1.00	0.905 (0.600, 1.365)	0.681 (0.450, 1.031)	0.653 (0.431, 0.990) *	0.0198

Note: Model 1 sequentially included each of the 14 trace elements individually, without adjustment for confounding factors. Model 2 sequentially included 14 trace elements with adjustment for confounding factors (age, sex, smoking, alcohol drinking, BMI, and hypertension). Model 3 further adjusted for education level and physical activity based on Model 2. Adjusted odds ratios (ORs) and 95% confidence intervals (CIs) were calculated for each interquartile range (IQR) increase in log-transformed values. Asterisks (*) indicate *p* < 0.05. *p* for trend denotes the presence of a linear increasing or decreasing trend in ORs across quartile increments.

## Data Availability

The datasets generated and/or analyzed during the current study are available from the corresponding author upon reasonable request.

## Supplementary Materials

The following supporting information can be downloaded at: https://www.mdpi.com/article/10.3390/nu18183068/s1, Table S1. Percentage of trace elements below the Limits of detection (LOD). Note: N (Below_LOD) indicates the number of samples with concentrations below the LOD; Percent (Below_LOD) indicates the percentage of samples below the LOD; Table S2. LASSO regression coefficients for the 14 selected trace elements. Note: Positive and negative values indicate the direction of association with T2DM; Table S3. Sensitivity analysis for the association between V and T2DM after removing physical activity from Model 3. Note: * *p* < 0.05. Estimates were adjusted for age, sex, smoking, alcohol drinking, BMI, hypertension, and education level.; Table S4. Association of 14 trace elements with fasting plasma glucose (FPG) risk assessed by multivariable linear regression. Note: In Model 1, each trace element was entered individually into a separate linear regression model. Model 2 was further adjusted for age, sex, smoking, alcohol drinking, BMI, hypertension, education level, and physical activity. Beta coefficients and their 95% CIs were calculated per interquartile range (IQR) increment of the logtransformed value. * *p* < 0.05. *p* for trend denotes whether the β coefficient shows a linear increasing or decreasing trend across quartiles; Table S5. Mean weights of the 14 trace elements in the WQS regression model. Note: Weights represent the relative contribution of each trace element to the mixture association with T2DM.; Table S6. BKMR interaction contrast estimates for V with Al, B, Li, Pb, and Te. Note: *p* (I > 0) denotes the posterior probability that the contrast estimate is greater than zero. Figure S1. Flow diagram of participant selection and matching process.
