# Supplementary material for: Serum Levels of 18 Trace Elements and Type 2 Diabetes Mellitus: A Propensity Score-Matched Case–Control Study in Northwest Chinese Adults
Source: Nutrients. 2026 Sep 20;18(18):3068. doi: 10.3390/nu18183068 (PMC13610063; doi:10.3390/nu18183068)
Supplement: Supplementary file 1 [file nutrients-18-03068-s001.zip › nutrients-4528239-supplementary.pdf]

Supplementary Materials

The supplementary materials for this study consist of six supplementary tables and one supplementary figure, as follows.

Table S1 Percentage of trace elements below the Limits of detection (LOD)

Element	N (Below LOD)	Percent (Below LOD)
Li	0	0
V	0	0
Fe	0	0
Co	0	0
Cu	0	0
Zn	0	0
Ba	0	0
B	0	0
Al	0	0
Cr	0	0
Mn	0	0
Ni	0	0
Se	0	0
Sr	0	0
Mo	0	0
Cd	7	0.55
Te	78	6.12
Pb	0	0

Note: N (Below LOD) indicates the number of samples with concentrations below the LOD; Percent (Below LOD) indicates the percentage of samples below the LOD.

Table S2 LASSO regression coefficients for the 14 selected trace elements.

Variable	Coefficient
Mo	-0.0362
V	-0.2883
B	-0.0016
Te	0.5603
Se	0.0034
Pb	-0.0155
Sr	-0.0026
Li	0.0342
Cr	-0.009
Ba	0.0041
Ni	-0.0017
Cu	3.00E-04
Al	4.00E-04
Mn	-0.0105

Note: Positive and negative values indicate the direction of association with T2DM.

Table S3 Sensitivity analysis for the association between V and T2DM after removing physical activity from Model 3.

Variable	continuous	Q1	Q2	Q3	Q4	P for trend
	O R(95%CI)		OR (95%CI)	OR (95%CI)	OR (95%CI)	
V	0.73(0.57,0.97) *	1.00	0.68(0.46,1.02)	0.73(0.48,1.10)	0.52(0.34,0.80)*	0.007

Note: *P < 0.05. Estimates were adjusted for age, sex, smoking, alcohol drinking, BMI, hypertension, and education level.

Table S4 Association of 14 trace elements with fasting plasma glucose (FPG) risk assessed by multivariable linear regression

Variable	continuous	Q1	Q2	Q3	Q4	P for trend
	β (95%CI)		β (95%CI)	β (95%CI)	β (95%CI)	
Li						
Model1	0.379(0.067,0.691) *	1.00	0.150(-0.471,0.771)	0.184(-0.437,0.805)	0.956(0.335,1.576) *	0.004
Model2	0.341(0.056,0.626) *	1.00	0.137(-0.428,0.702)	0.118 (-0.448,0.684)	0.818 (0.252,1.385) *	0.008
B						
Model1	-0.444(-0.646, -0.242) *	1.00	-1.141(-1.761, -0.522) *	-0.835(-1.455, -0.215) *	-0.858(-1.477, -0.239) *	0.024
Model2	-0.315(-0.500, -0.129) *	1.00	-0.596(-1.167, -0.024) *	-0.476(-1.044,0.092)	-0.371(-0.942,0.201)	0.281
Al						
Model1	0.020(-0.159,0.199)	1.00	-0.163 (-0.786,0.460)	0.249 (-0.374,0.872)	-0.083(-0.705,0.540)	0.871
Model2	-0.016(-0.181,0.148)	1.00	-0.176(-0.743,0.391)	0.098 (-0.471,0.668)	-0.254(-0.825,0.317)	0.599
Se						
Model1	0.706(-0.033,1.445)	1.00	0.270(-0.352,0.891)	-0.094(-0.716,0.528)	0.644(0.023,1.265) *	0.118
Model2	0.341(-0.339,1.021)	1.00	0.324(-0.242,0.891)	-0.202(-0.772,0.368)	0.362 (-0.208,0.932)	0.537
Sr						
Model1	0.747(-0.012,1.507)	1.00	-0.267(-0.889,0.354)	-0.296(-0.917,0.326)	0.507(-0.114,1.128)	0.137
Model2	0.256(-0.452,0.945)	1.00	-0.248(-0.815,0.319)	-0.374(-0.947,0.198)	0.145(-0.431,0.721)	0.743
V						
Model1	-0.033(-0.286,0.219)	1.00	0.419(-0.582,0.665)	0.099(-0.525,0.722)	0.188(-0.435,0.811)	0.536
Model2	-0.261(-0.497, -0.025) *	1.00	-0.108(-0.677,0.461)	-0.269(-0.846,0.309)	-0.375(-0.958,0.207)	0.173
Te						
Model1	0.126(-0.121,0.373)	1.00	-0.103(-0.725,0.520)	-0.062(-0.684,0.560)	0.521(-0.101,1.143)	0.109
Model2	0.102(-0.123,0.327)	1.00	0.074(-0.494,0.642)	0.120(-0.447,0.687)	0.470(-0.099,1.04)	0.113
Pb						
Model1	-0.312(-0.490, -0.134) *	1.00	-0.252(-0.874,0.370)	-0.818(-1.440, -0.197) *	-0.678(-1.199,0.043)	0.022
Model2	-0.198(-0.361, -0.034) *	1.00	-0.078(-0.645,0.489)	-0.622(-1.190, -0.055) *	-0.232(-0.801,0.338)	0.178
Ba						
Model1	0.034(-0.198,0.265)	1.00	0.210(-0.412,0.832)	0.729(0.107,1.351) *	0.386(-0.236,1.007)	0.095
Model2	0.043(-0.172,0.259)	1.00	0.373 (-0.194,0.941)	0.809 (0.240,1.378) *	0.355(-0.224,0.934)	0.097
Cr						

Model1	-0.033(-0.212,0.147)	1.00	-0.174(-0.798,0.449)	-0.145(-0.768,0.479)	-0.199(-0.822,0.424)	0.571
Model2	-0.075(-0.238,0.088)	1.00	0.001(-0.567,0.568)	-0.031(-0.599,0.536)	-0.331(-0.899,0.236)	0.263
Cu						
Model1	0.979(0.178,1.779) *	1.00	0.375(-0.246,0.997)	0.240(-0.382,0.861)	0.922(0.301,1.543) *	0.009
Model2	0.908(0.167,1.648) *	1.00	0.632(0.062,1.203) *	0.363(-0.209,0.936)	1.001(0.424,1.578) *	0.003
Mn						
Model1	0.028(-0.190,0.246)	1.00	0.17(-0.453,0.793)	0.232(-0.391,0.856)	0.217(-0.406,0.839)	0.478
Model2	0.145(-0.185,0.214)	1.00	0.183(-0.385,0.751)	0.211(-0.357,0.778)	0.134(-0.435,0.702)	0.638
Mo						
Model1	0.201(-0.171,0.573)	1.00	-0.261(-0.884,0.361)	-0.055(-0.678,0.568)	0.296(-0.326,0.919)	0.275
Model2	0.160(-0.181,0.502)	1.00	-0.367(-0.934, -0.201)	-0.139(-0.709,0.431)	0.049(-0.526,0.624)	0.693
Ni						
Model1	-0.086(-0.253,0.081)	1.00	-0.109(-0.732,0.513)	-0.496(-1.119,0.127)	-0.217(-0.839,0.406)	0.302
Model2	-0.083(-0.236,0.069)	1.00	-0.259(-0.826,0.309)	-0.484(-1.053,0.085)	-0.271(-0.838,0.295)	0.257

Note: In Model 1, each trace element was entered individually into a separate linear regression model. Model 2 was further adjusted for age, sex, smoking, alcohol drinking, BMI, hypertension, education level, and physical activity. Beta coefficients and their 95% CIs were calculated per interquartile range (IQR) increment of the log-transformed value. *$P < 0.05$. P for trend denotes whether the β coefficient shows a linear increasing or decreasing trend across quartiles.

Table S5 Mean weights of the 14 trace elements in the WQS regression model.

mix name	mean weight
Mo	0.286087
V	0.20069
B	0.130421
Te	0.080694
Se	0.07861
Pb	0.060452
Sr	0.055159
Li	0.049365
Cr	0.025239
Ba	0.022111
Ni	0.009283
Cu	0.001766
Al	0.00012
Mn	1.58E-06

Note: Weights represent the relative contribution of each trace element to the mixture association with T2DM.

Table S6 BKMR interaction contrast estimates for V with Al, B, Li, Pb, and Te.

Interaction pair	Contrast estimate	Posterior SD	Approx.95% posterior interval	P(I>0)
V × Al	-0.00007	0.02009	[-0.0395, 0.0393]	0.499
V × B	-0.00152	0.01092	[-0.0229, 0.0199]	0.445
V × Li	-0.00910	0.02851	[-0.0650, 0.0468]	0.375
V × Pb	0.00040	0.01560	[-0.0302, 0.0310]	0.510
V × Te	-0.00375	0.00978	[-0.0229, 0.0154]	0.351

Note: *P*(I>0) denotes the posterior probability that the contrast estimate is greater than zero.

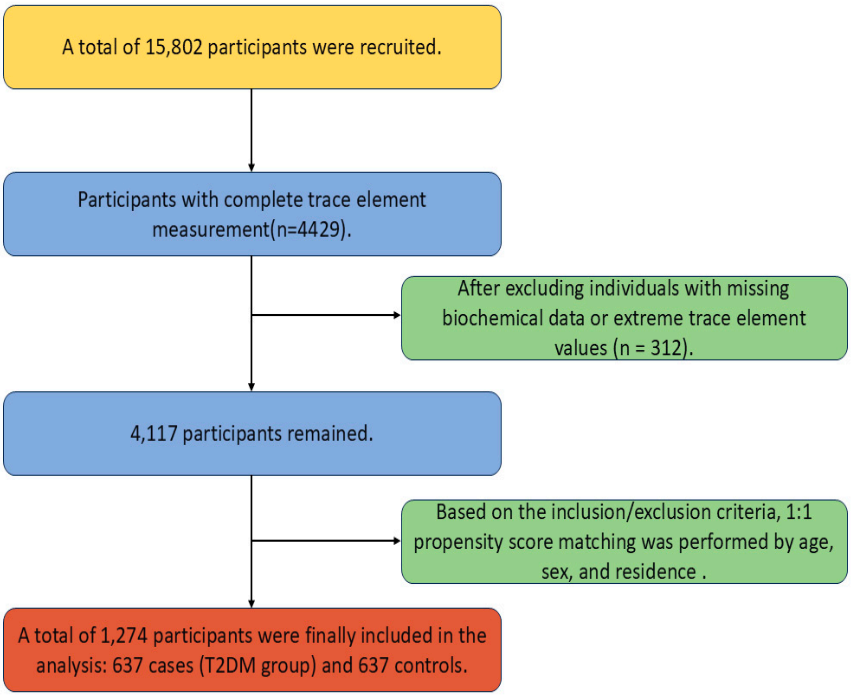

Figure S1. Flow diagram of participant selection and matching process
